# PEG Linker Improves Antitumor Efficacy and Safety of Affibody-Based Drug Conjugates

**DOI:** 10.3390/ijms22041540

**Published:** 2021-02-03

**Authors:** Qiyu Li, Wenjing Li, Keyuan Xu, Yutong Xing, Haobo Shi, Zhe Jing, Shuang Li, Zhangyong Hong

**Affiliations:** State Key Laboratory of Medicinal Chemical Biology, College of Life Sciences, Nankai University, Tianjin 300071, China; qiyuli@mail.nankai.edu.cn (Q.L.); L800450@163.com (W.L.); 1120190518@mail.nankai.edu.cn (K.X.); xingyutong@mail.nankai.edu.cn (Y.X.); 2120191069@mail.nankai.edu.cn (H.S.); jznku@mail.nankai.edu.cn (Z.J.)

**Keywords:** HER2, affibody, antibody drug conjugates (ADCs), MMAE

## Abstract

Antibody drug conjugates (ADCs) have become an important modality of clinical cancer treatment. However, traditional ADCs have some limitations, such as reduced permeability in solid tumors due to the high molecular weight of monoclonal antibodies, difficulty in preparation and heterogeneity of products due to the high drug/antibody ratio (4–8 small molecules per antibody). Miniaturized ADCs may be a potential solution, although their short circulation half-life may lead to new problems. In this study, we propose a novel design strategy for miniaturized ADCs in which drug molecules and small ligand proteins are site-specifically coupled via a bifunctional poly(ethylene glycol) (PEG) chain. The results showed that the inserted PEG chains significantly prolonged the circulation half-life but also obviously reduced the cytotoxicity of the conjugates. Compared with the conjugate Z_HER2_-SMCC-MMAE (HM), which has no PEG insertion, Z_HER2_-PEG4K-MMAE (HP4KM) and Z_HER2_-PEG10K-MMAE (HP10KM) with 4 or 10 kDa PEG insertions have 2.5- and 11.2-fold half-life extensions and 4.5- and 22-fold in vitro cytotoxicity reductions, respectively. The combined effect leads to HP10KM having the most ideal tumor therapeutic ability at the same dosages in the animal model, and its off-target toxicity was also reduced by more than 4 times compared with that of HM. These results may indicate that prolonging the half-life is very helpful in improving the therapeutic capacity of miniaturized ADCs. In the future, the design of better strategies that can prolong half-life without affecting cytotoxicity may be useful for further improving the therapeutic potential of these molecules.

## 1. Introduction

Human epidermal growth factor receptor 2 (HER2), also known as erbB-2, is a receptor tyrosine-protein kinase that belongs to the epidermal growth factor receptor family [1,2]. It is expressed at low levels in normal tissues but is expressed highly in many tumor tissues, such as breast, ovarian, prostate, and several gastric cancers [3], which makes it an effective target for tumor therapy [4]. Some monoclonal antibodies against HER2, such as trastuzumab and pertuzumab, have been successfully developed for the treatment of cancers associated with HER2 overexpression. Compared with traditional monoclonal antibodies, monoclonal antibody drug conjugates (ADCs) can significantly improve the killing ability of the antibody through coupling with highly active small molecules. At present, some anti-HER2 ADCs, such as Kadcyla (ado-trastuzumab emtansine) [5,6] and Enhertu (fam-trastuzumb deruxtecan-nxki) [7,8], have also been successfully developed, and they have significantly improved the survival rate and prognosis of patients [5,9].

Nevertheless, the current ADC strategy has some limitations. Conventional ADC drugs use large monoclonal antibodies (~150 kDa), such as trastuzumab, as tumor-targeting ligands, whose huge molecular structure reduces the efficiency of drug molecules penetrating solid tumor tissues and limits the therapeutic effectiveness of this strategy in solid tumor treatment [10,11]. In addition, due to the large molecular weight of monoclonal antibodies (~150 kDa), multiple small molecule drugs (4–8 small molecules) per antibody molecule need to be coupled to achieve satisfactory tumor killing ability, which makes the preparation and site-specific connection of drugs very difficult. Moreover, the synthesized product molecules are often complex mixtures of multiple positional isomers with varied numbers of small molecules [12,13], which makes purification and quality control difficult. To circumvent these limitations, new strategies for ADC drug design need to be developed.

Using antibody fragments, such as single-chain variable fragments (scFvs, ~28 kDa) [14], Fabs (~54 kDa) [15,16], diabodies (~50 kDa) [17,18], and nanobodies (~14 kDa), or miniaturized antibody analogs, such as designed ankyrin repeat proteins (DARPins) [19,20,21] and affibodies [22,23], to replace full-length antibodies for ADC synthesis is expected to compensate for the above problems. Among them, affibody molecules are extremely small proteins with only 58 amino acids, and they are simply composed of three helical bundles and have many attractive physical or chemical properties, such as high stability and easy preparation [24,25,26]. Moreover, various high-throughput screening technologies are available for rapid structural modification and affinity enhancement. These advantages make affibodies very attractive for biological diagnostic and therapeutic applications. Among them, Z_HER2:342_ and its derivative Z_HER2:2891_ are two anti-HER2 affibody molecules that have extremely high binding capacity towards the HER2 receptor, with equilibrium dissociation constant (K_D_) of 22 and 60 pM, respectively [27]. In previous reports, these molecules were used in tumor diagnosis by coupling with radioisotope radioactive isotopes, such as ^99m^Tc, ^18^F, and ^111^In, and some of them are currently being tested in clinical trials [28,29,30]. Moreover, affibody molecules do not contain any cysteine residues; therefore, site-specific coupling with small molecule drugs can be realized by introducing an extra cysteine residue [23,31]. Therefore, constructing miniaturized ADCs by using affibody molecules as tumor-targeting ligands is a potentially useful strategy [32].

However, compared with conventional full-size monoclonal antibody-based ADC drug molecules, affibody-based conjugates may have the problem of short in vivo circulation half-life, which may limit the drug accumulation efficiency in tumor sites and in turn affect the tumor therapeutic ability of the drugs [33]. How to prepare affibody-based miniaturized ADC molecules by overcoming their circulation half-life problem may be very important for improving the application potential of this strategy. PEGylation is a practical and effective strategy to improve the half-life of protein drugs in vivo [34,35]. However, PEGylation may reduce the cytotoxicity of the conjugates while increasing the half-life, where the degree of influence may be closely related to the molecular weight of the poly(ethylene glycol) (PEG) chain [36,37,38]. In addition, the coupling mode of PEG chains and small drug molecules may affect affibody affinity with their targets and then in turn affect the biological activity of the conjugates [39,40]. Therefore, exploring the appropriate length of PEG chains and appropriate strategies for coupling PEG chains, small molecule drugs, and affibody molecules to ensure the effective half-life extension and ideal cytotoxicity of the conjugates, the simplicity of the preparation and the maximum maintenance of the affibody affinity are crucial for the application of the strategy [41].

**Figure 1:**
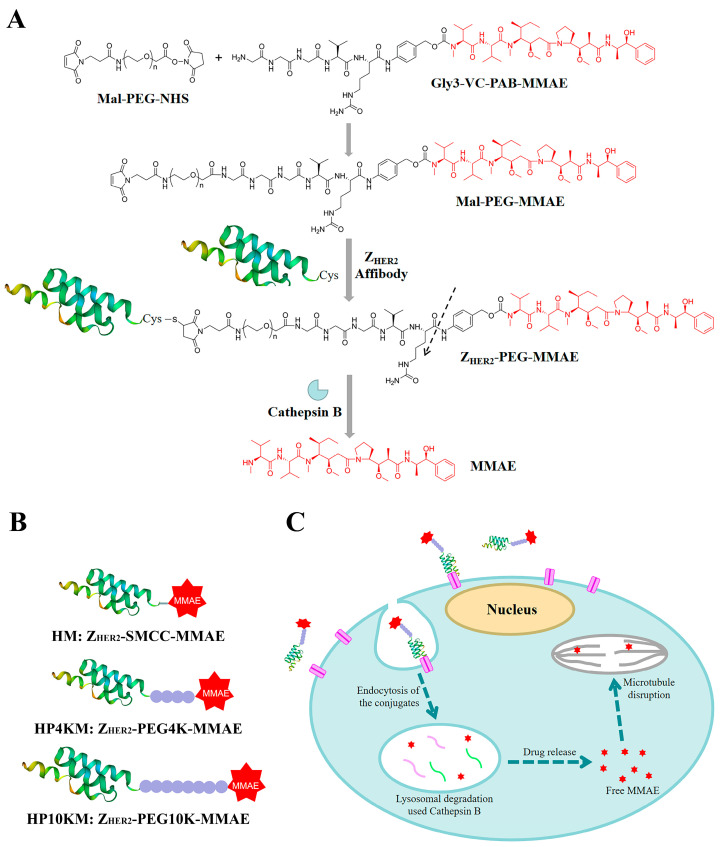
Strategy of comodification of affibody Z_HER2:2891_ with PEG chains and MMAE. (**A**) Structure of Gly3-VC-PAB-MMAE, synthetic strategy for generation of the different conjugates and cathepsin B drug release. (**B**) Schematic illustration of HM, HP4KM, and HP10KM. (**C**) General mechanism of the action of the conjugates. The conjugates bind to their target receptor (HER2 protein) on the cell surface to form an ADC-antigen complex, leading to endocytosis of the complex. The internalized conjugates undergo lysosomal processing and trigger intracellular drug release, leading to tumor cell death.

This study explores a simple strategy for the preparation of affibody-based miniatured ADCs by using PEG chains with bifunctional groups as linkers to achieve site-specific coupling of small molecule drugs and affibody proteins and simultaneously improve the circulation half-life (Figure 1). Here, we chose the affibody Z_HER2:2891_ as the target molecule, which has excellent binding affinity with the HER2 receptor and relatively better hydrophilicity and structural stability than Z_HER2:342_ [32]. A cysteine residue was introduced into its C-terminus, and the reaction between the sulfhydryl group and maleimide group and between the activated ester and amino group were used to realize the site-specific coupling of the affibody protein, PEG chain and small molecular drug monomethyl auristatin E (MMAE). MMAE is a tubulin inhibitor [42,43] that was used as the toxic moiety, and its N-terminus was linked with a specific valine-citrulline-PAB (Val-Cit-PAB) sequence, which can be selectively cleaved by cathepsin B in lysosomes to release MMAE in tumor cells [14,42,44]. In this study, we synthesized three affibody-based conjugates, i.e., Z_HER2_-SMCC-MMAE (HM), Z_HER2_-PEG4K-MMAE (HP4KM) and Z_HER2_-PEG10K-MMAE (HP10KM), with varied molecular weights of PEG modification, and evaluated their circulation half-lives and in vitro and in vivo antitumor effects in detail.

Finally, we found that HP10KM has the most ideal tumor therapeutic ability compared with the other two conjugates. In this molecule, the modification of the 10 kDa PEG chain reduces the cytotoxicity but significantly prolongs the circulation half-life of this conjugate, and the combined results lead to a stronger tumor growth inhibition effect in the NCI-N87 tumor model. The results may well demonstrate that the prolongation of half-life is very helpful for improving the therapeutic ability of affibody-based ADC drugs, even if the activity is impaired. In the future, if we can find better strategies to prolong the half-life and maintain the cytotoxicity of the miniature conjugates at the same time, it is possible to further improve the therapeutic capability of these conjugates.

## 2. Results and Discussion

### 2.1. Synthesis of the Conjugates

We designed and prepared three anti-HER2 affibody Z_HER2:2891_-based conjugates, namely, HM, HP4KM, and HP10KM (Figure 1). In these molecules, Z_HER2:2891_ and MMAE are located at the ends and connected by succinimidyl-trans-4-(N-maleimidylmethyl)cyclohexane-1-carboxylate (SMCC) or PEG chains with bifunctional groups, i.e., maleimide and activated ester, which can react specifically and efficiently with sulfhydryl and amine groups. The PEG chain (or SMCC) is located in the middle of the conjugates. A cysteine residue was introduced at the C-terminus of Z_HER2:2891_ for site-specific coupling based on the reaction between the sulfhydryl group and maleimide group. The amine group in the (glycine)3-citruline-p-amino-benzyloxycarbonyl-monomethyl auristatin E (Gly3-VC-PAB-MMAE) molecule can react specifically with the activated ester group at the other end of the bifunctional SMCC or PEG chain to realize the connection of the three parts.

**Figure 2:**
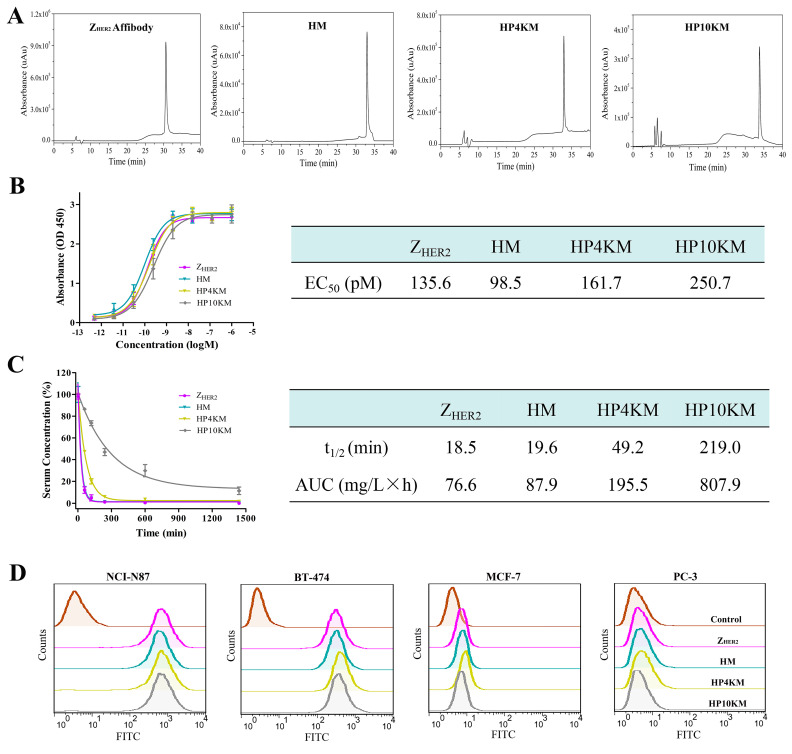
HPLC characterization and determination of the affinity and half-life of the HM, HP4KM, and HP10KM conjugates. (**A**) HPLC analysis of affibody Z_HER2:2891_ and the conjugates on a C-4 column. (**B**) Binding analysis of the conjugates towards HER2 receptors by ELISA. (**C**) Circulation clearance of the conjugates determined by ELISA. Serum samples were collected from BALB/c mice injected with the conjugates at different time points post injection, and the concentration of the residual conjugates in the plasma was determined by ELISA. t1/2: half-life. AUC: area under the curve from zero to infinity. (**D**) Binding specificity of the conjugates detected by flow cytometry. Data were expressed as the mean ± SEM (*n* = 4).

The affibody Z_HER2:2891_ with a cysteine residue at the C-terminus and a 6 × His Tag at the N-terminus was expressed in the soluble fraction in the *Escherichia coli* system. High-performance liquid chromatography (HPLC, Figure 2A) and sodium dodecyl sulfate polyacrylamide gel electrophoresis (SDS-PAGE, Figure S1) analysis showed that the purified protein had a purity higher than 95% after simple nickel column purification. However, the free sulfhydryl group in the protein easily forms disulfide bonds during purification and storage, and these bonds need to be reduced with tris (2-carboxyethyl)-phosphine (TCEP) before coupling with a maleimide group.

The assembly of Z_HER2:2891_, Gly3-VC-PAB-MMAE, and SMCC or PEG chains was achieved through a two-step operation. First, the bifunctional SMCC or PEG chains were coupled with Gly3-VC-PAB-MMAE via the reaction between the activated ester and amino group in the presence of DIPEA as the catalyst. The efficiency of this reaction is very high, and complete coupling is achieved under approximately 1:1.2 equivalents of SMCC or PEG chains and Gly3-VC-PAB-MMAE. Without any purification, the product was directly coupled with the reduced Z_HER2:2891_ through the reaction between free sulfhydryl and maleimide. The coupling was also achieved completely in the case of 1:1.1 equivalents of the two reactants. The crude products were subjected to Ni column purification, and the conjugates HM, HP4KM, and HP10KM were obtained with purities higher than 95%, as verified by HPLC (Figure 2A). MALDI-TOF mass spectrometry (Figures S2–S5) corroborated the identities of HM (10,092.69 Da; theoretical molecular mass, 10,101.1 Da), HP4KM (13,765.90 Da; theoretical molecular mass, 13,782.97 Da) and HP10KM (19,721.93 Da; theoretical molecular mass, 19,782.97 Da).

### 2.2. Receptor Binding Analysis Based on Enzyme Linked Immunosorbent Assay (ELISA)

ELISA was used to determine the binding affinity between these conjugates and the HER2 receptor, and the results are shown in Figure 2B. The results showed that within a certain concentration range, the light absorption intensity gradually increased as the conjugate concentration increased. The calculated EC_50_ values of Z_HER2:2891_, HM, HP4KM, and HP10KM with HER2 protein are 135.6, 98.5, 161.7, and 250.7 pM, respectively (Figure 2B).

These results indicate that these conjugates have extremely high affinity for their receptor HER2. Moreover, when MMAE or PEG chains with even larger molecular weights were coupled to the Z_HER2:2891_ protein, there was no significant difference between the EC_50_ values of these conjugates and Z_HER2:2891_ with the HER2 receptor. The drug or PEG chains were chosen to be coupled at the C-terminus of Z_HER2:2891_, which is far from its receptor binding region. This design may help to reduce the influence of conjugation on the affinity of affibody molecules. The high binding affinity of these molecules with the HER2 receptor lays a good foundation for their specific and efficient killing of HER2-overexpressing tumors.

### 2.3. Half-Life Extension of the Conjugates

The in vivo blood circulation half-life of these conjugates was determined by quantification of the residual proteins in plasma with ELISA and then calculation of the clearance rate of the conjugates with GraphPad Prism 6.0 software. As shown in Figure 2C, the half-life of HM was only 19.6 min, which is similar to that of Z_HER2:2891_ (18.5 min). After insertion of long-chain PEG with a molecular weight of 4 or 10 kDa, the half-lives of the HP4KM and HP10KM molecules were extended to 49.2 and 219.0 min, respectively, which are equal to 2.5-fold and 11.2-fold extensions, respectively.

HM molecules without PEG chains have a very short half-life of 19.6 min and should have limited efficiency to accumulate in tumor tissue for rapid clearance from the body. When a 4 or 10 kDa PEG chain was used as the linker between MMAE and the affibody ligand, the half-life was significantly improved. Insertion of a PEG chain between the drug and affibody truly can significantly improve the half-life of the conjugates. However, it is worth mentioning that modification with a 4 kDa PEG chain could only extend the half-life of the conjugate by 2.5-fold, while modification with a 10 kDa PEG chain could extend the half-life by 11.2-fold. PEGylation can increase the molecular volume of conjugates, thus reduce the probability of being filtered out by the kidney and then prolong the circulation half-life of the conjugates in the body. In a certain range, with the increase of PEG molecular weight, the elongation effect will be further strengthened. This result indicated that selecting PEG chains with appropriate molecular weights may be very important to achieve satisfactory half-life extension.

### 2.4. Binding Selectivity Analyzed by Flow Cytometry

Flow cytometry was adopted to further determine the binding affinity and selectivity of these conjugates with the HER2 receptor. Human gastric cancer NCI-N87 cells and human breast cancer BT-474 cells with high HER2 receptor expression, human breast cancer MCF-7 cells with low HER2 receptor expression, and human prostatic carcinoma PC-3 cells with almost negative HER2 receptor expression were used for analysis. As shown in Figure 2D, all conjugates had a very strong interaction with the HER2-positive cell lines NCI-N87 and BT-474, with much stronger fluorescence intensity than the control group. However, for the HER2-low expression cell line MCF-7 and the negative cell line PC-3, there were no significant differences in fluorescence intensity between the experimental group and the control group. These results indicated that these conjugates have good binding affinity and target selectivity towards the HER2 receptor and also showed that the coupling of MMAE and long PEG chains did not affect the binding affinity of the conjugates.

### 2.5. In Vitro Cytotoxicity of the Conjugates

**Figure 3:**
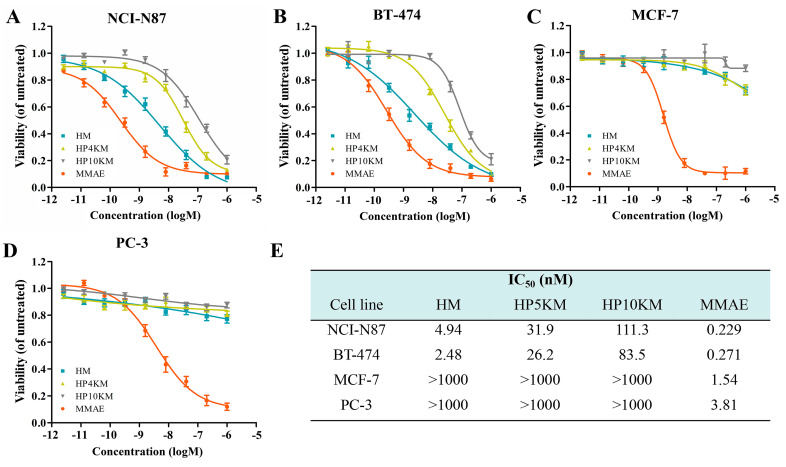
Cell viability determined using MTT assays. NCI-N87 cells (**A**), BT-474 cells (**B**), MCF-7 cells (**C**), and PC-3 cells (**D**) were incubated with different concentrations of HM, HP4KM, HP10KM, or MMAE for 72 h, and then, the cell viability was determined through MTT assays. (**E**) IC_50_ data of the conjugates HM, HP4KM, HP10KM, and MMAE against various tumor cell lines. Data were expressed as the mean ± SEM (*n* = 5).

The cytotoxic activity of these conjugates was measured by a 3-(4,5-dimethylthiazol-2-yl)-2,5-diphenyltetrazolium bromide (MTT) assay, which is a commonly used method to determine cell viability. The living cells in the culture medium transformed MTT reagent into formazan, whose concentration is proportional to the number of the surviving cells post treatment. IC_50_ values (half of the maximum inhibitory concentration) were used to predict the degree of the cytotoxicity of the conjugates [45,46]. HER2 receptor-positive NCI-N87 and BT-474 cells, MCF-7 cells with low HER2 receptor expression, and HER2 receptor-negative PC-3 cells in the log phase of growth were incubated with various concentrations of these conjugates (2.56 pM to 1.0 μM), and cell viabilities were measured by MTT assay. Free MMAE was used as a control. As shown in Figure 3, the affibody-based conjugates, including HM, HP4KM, and HP10KM, all showed very strong cytotoxic activity towards HER2-positive cells, and their IC_50_ values ranged from 4.0 to 100 nM. Among them, HM that directly coupled MMAE with Z_HER2:2891_ showed the strongest activity, and its IC_50_ values towards HER2-positive NCI-N87 and BT-474 cells were 4.94 and 2.48 nM, respectively. HP4KM modified with a 4 kDa PEG chain has IC_50_ values of 31.9 and 26.2 nM towards these two cell lines, while HP10KM coupled with a 10 kDa PEG chain has IC_50_ values of 111.3 and 83.5 nM towards them, respectively. In comparison, the intercalated PEG chain has a negative effect on the cytotoxicity of the conjugates. For HER2-low or HER2-negative MCF-7 or PC-3 cells, all conjugates showed much reduced cytotoxicity, with IC_50_ values higher than 1000 nM, which is almost 50-fold higher than those towards HER2-positive cells. These data indicate that these conjugates can kill HER2-positive cancer cells in a highly selective manner dependent on the HER2 receptor. In contrast, free MMAE molecules showed very strong killing ability towards HER2-negative or HER2-positive cells but had no obvious targeting selectivity, with IC_50_ values towards these cells in the range of 0.229~3.81 nM. A lack of necessary selectivity of cytotoxicity would cause strong side effects, which is also the main reason MMAE cannot be used in the clinic.

**Figure 4:**
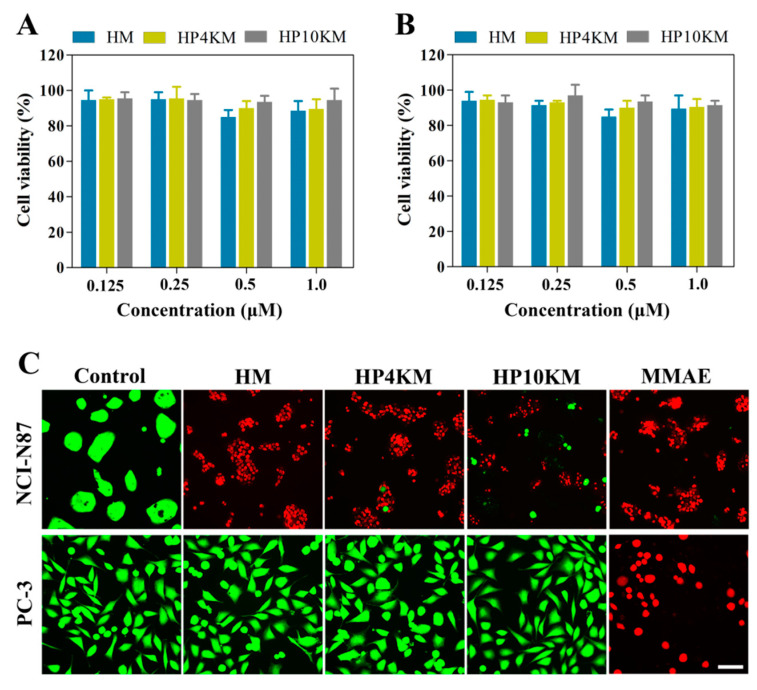
Competitive inhibition assay and live/dead staining imaging for detection of the cytotoxicity of the conjugates. NCI-N87 cells (**A**) and BT-474 cells (**B**) were preincubated with the affibody Z_HER2:2891_ (5 µM) for 2 h before the conjugates were added, and the cell viability was determined by in vitro cytotoxicity assays. (**C**) NCI-N87 cells and PC-3 cells were incubated with 200 nM of the conjugates for 72 h and then visualized by microscopy imaging. Scale bars, 20 μm.

To further verify the role of the HER2 receptor in the cytotoxicity of these conjugates, a competitive inhibition assay was used by preincubating cells with an excess of Z_HER2:2891_ (50 μM, for 2 h) to block the HER2 receptor. Here, the cytotoxicity of the conjugates was significantly reduced towards HER2 receptor-positive tumor cells, including NCI-N87 and BT-474 cells. Under these conditions, the survival rate of HER2-positive cells was still above 90%, even when the concentration of conjugates was increased to 1.0 μM (Figure 4A,B). These results demonstrated that the cytotoxicity of these conjugates towards cells is highly dependent on the expression of the HER2 receptor on the cell surface.

The above results indicate that these molecules all have strong cytotoxic activity and excellent HER2 receptor-dependent selectivity. However, long-chain PEG modification has a negative effect on the cytotoxicity of the conjugates. Modification with 4 or 10 kDa PEG chains reduced the cytotoxicity of the conjugates by approximately 6.5- and 22.5-fold, respectively. However, both HP4KM and HP10KM still have excellent cytotoxicity. Moreover, modification with long PEG chains did not change the selectivity and receptor dependence of the conjugates.

### 2.6. Microscopy Analysis of the Cytotoxicity of the Conjugates

Live/dead staining was used to further visualize the cytotoxic activity of these conjugates. HER2-positive NCI-N87 cells and HER2-negative PC-3 cells were spread on 96-well plates and incubated with 200 nM HM, HP4KM, HP10KM, and MMAE. After incubation for 72 h, the cells were stained with Calcein-AM (green) and EthD-1 (red) and observed with a microscope. As shown in Figure 4C, when HER2-positive NCI-N87 cells were coincubated with these compounds, 200 nM HM almost completely killed all cells, with almost no green signals specific to living cells; and HP4KM and HP10KM could kill most of the cells, with only a few green signals of living cells remaining. Compared with HM, the cytotoxicity of HP4KM and HP10KM decreased due to conjugation with the long PEG chain. For HER2-negative PC-3 cells, coincubation with HM, HP4KM, and HP10KM did not cause the death of PC-3 cells, with almost all cells showing a green signal from living cells. In contrast, free MMAE killed almost all HER2-positive NCI-N87 cells and HER2-negative PC-3 cells, showing no selectivity. These results indicate that HM, HP4KM, and HP10KM have strong cytotoxicity against HER2-positive cells and good selectivity for the HER2 receptor. With the modification of the PEG chain, the cytotoxic activity of the conjugate decreased. However, free MMAE did not have cytotoxicity selectivity.

### 2.7. Apoptotic Mechanism of the Conjugates

**Figure 5:**
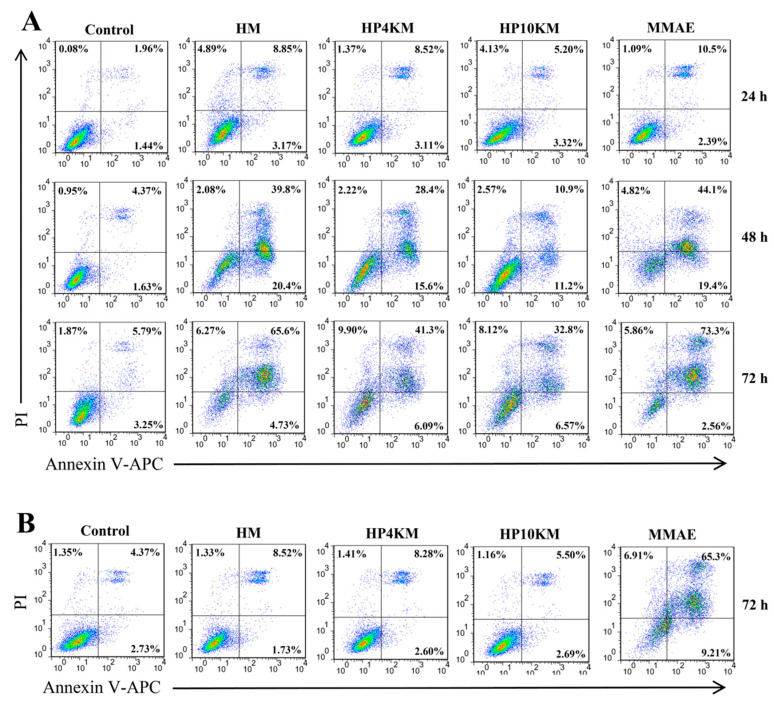
Apoptotic mechanism of the conjugates analyzed by flow cytometry. HER2-positive NCI-N87 cells (**A**) were treated with 200 nM of the conjugates for 24, 48, and 72 h, or HER2-negative PC-3 cells (**B**) were treated with 200 nM of the conjugates for 72 h, stained with Annexin V-APC/PI and analyzed for apoptosis by flow cytometry.

Annexin V-APC/PI staining was used to analyze the effects of the PEG chain and MMAE on apoptosis induced by these molecules. As shown in Figure 5, HM, HP4KM, and HP10KM could induce significant apoptosis signals when HER2-overexpressing NCI-N87 cells were incubated with these molecules. However, almost no effective apoptosis was induced when HER2-negative PC-3 cells were incubated with the molecules (Figure 5B). In contrast, free MMAE induced strong apoptosis in either HER2-positive NCI-N87 cells or HER2-negative PC-3 cells, with killing rates of 73.3% and 65.3%, respectively, showing no obvious selectivity.

In addition, the effect of incubation time on apoptosis was also determined. In NCI-N87 cells, the experimental group had almost no apoptosis signal when coincubated with HM, HP4KM, or HP10KM for 24 h. When the incubation time was extended to 48 h, the cells gradually entered the early apoptosis stage (AV^+^PI^+^). After 72 h of incubation, many early apoptotic cell groups entered the late apoptosis stage (AV^+^PI^+^). At this time, the apoptosis rates induced by HM, HP4KM, and HP10KM reached 65.6%, 41.3%, and 32.8%, respectively. For all incubation times, the proportion of apoptosis in the PBS group was less than 10% in any cell line.

The above results indicate that the conjugates HM, HP4KM, and HP10KM can significantly induce apoptosis in a HER2 receptor-dependent manner. With prolonged incubation time, the cells gradually transitioned from the early stage into the late stage of apoptosis. At the same time, modification with a long PEG chain in the conjugates has a certain negative effect on the induced apoptosis activity, and the longer the PEG chain is, the more obvious the effect.

### 2.8. Cell Cycle Arrest in HER2-Positive NCI-N87 Cells

**Figure 6:**
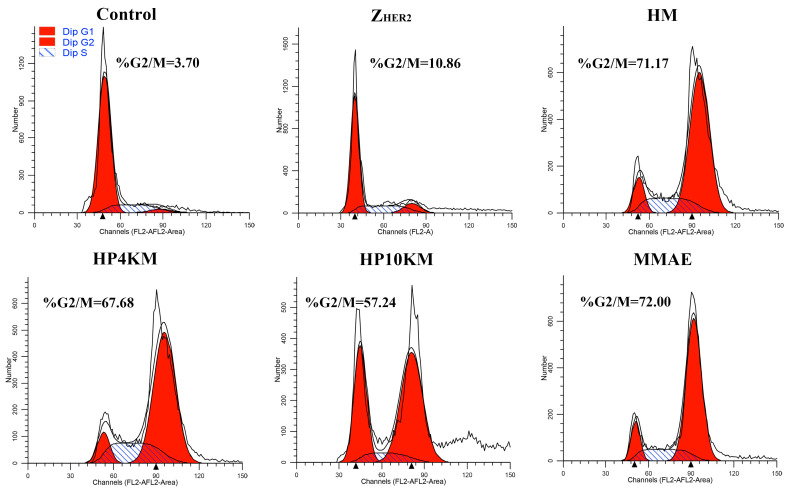
Selective induction of G2 growth arrest by these conjugates. NCI-N87 cells were exposed to 1.0 μM conjugates, 1.0 μM Z_HER2:2891_ affibody or free MMAE for 16 h before analysis by flow cytometry. %G2/M indicates the percentage of cells arrested at the G2/M phase.

Since MMAE inhibits cell division by inhibiting tubulin polymerization, we examined the effect of these conjugates and free MMAE on the cell cycle of NCI-N87 cells. Compared with the PBS control, MMAE strongly inhibited NCI-N87 cell division, and the percentages of cells in G2/M phase in the groups treated with PBS and MMAE were 3.7% and 72.0%, respectively (Figure 6). Similar to MMAE, the HM, HP4KM, and HP10KM conjugates also strongly inhibited cell division, and the percentages in G2/M phase of the cells treated with these conjugates were 71.2%, 67.7%, and 57.2%, respectively (Figure 6 and Figure S6). However, the ability of these conjugates to inhibit cell division also decreases with increasing molecular weight of the PEG chain. Free Z_HER2:2891_ has almost no ability to affect the cell cycle, with only 10.9% of the treated cells located in the G2/M phase, showing no significant difference from that of the PBS control group. These experimental results demonstrated that these conjugates conferred the capability of G2/M phase on NCI-N87 cells while PEG chain modification reduced the lethality of the conjugates.

### 2.9. In Vivo Efficacy of the Conjugates in an NCI-N87 Xenograft Model

In vivo tumor growth inhibition of these conjugates was evaluated in an NCI-N87 gastric cancer xenograft model. NCI-N87 cells (5.0 × 10^6^) were injected subcutaneously into BALB/c nude mice. When the tumors reached approximately 210 mm^3^, the mice were randomly grouped and treated with these conjugates at two different dosages (1.5 and 0.6 mg/kg) every three days for a total of four times on the day 1, 4, 7, and 11.

**Figure 7:**
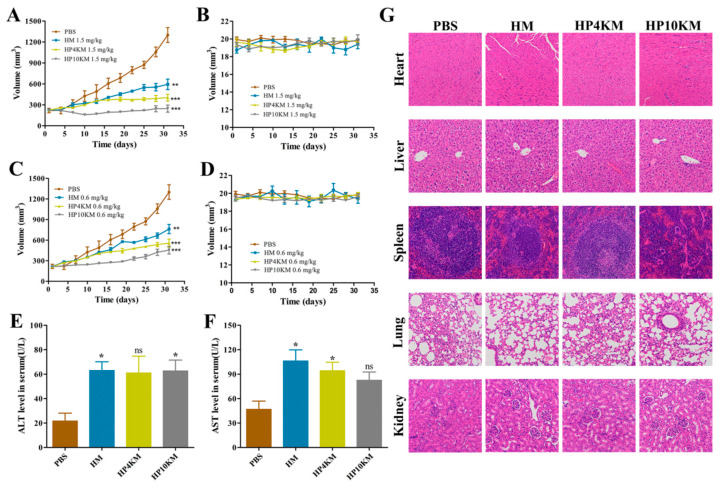
In vivo antitumor activity of these conjugates. Tumor growth curves (**A**) and body weight change (**B**) of NCI-N87 tumor-bearing BALB/c nude mice after intravenous administration of the conjugates at a dose of 1.5 mg/kg. Tumor growth curves (**C**) and body weight change (**D**) of NCI-N87 tumor-bearing BALB/c nude mice after intravenous administration of the conjugates at a dose of 0.6 mg/kg. ALT (**E**) and AST (**F**) in the serum of BALB/c nude mice receiving 1.5 mg/kg treatment were determined by ELISA after four treatments. (**G**) Histological analysis of major tissues after treatment with these conjugates (200×). Data were expressed as the mean ± SEM (*n* = 5). Statistical significance was calculated by Student’s *t*-test: * *p* < 0.05; ** *p* < 0.01; *** *p* < 0.001.

As shown in Figure 7, the tumors in the control group where the mice received the injection of PBS grew rapidly, with a mean tumor volume of 1000 mm^3^ on day 25 and 1300 mm^3^ on day 31 post first injection of the conjugates. In the treatment group, injection of HM, HP4KM, or HP10KM at doses of either 1.5 mg/kg or 0.6 mg/kg significantly inhibited tumor growth, although the therapeutic effect with the dosage of 1.5 mg/kg was more obvious. On day 31 post first injection of the conjugates, the tumor volumes of the HM, HP4KM, and HP10KM groups with this dosage were 593, 403, and 245 mm^3^, respectively, and the corresponding tumor inhibition rates were 54.38%, 69.00%, and 81.15%, respectively. The therapeutic effect with the dosage of 0.6 mg/kg was slightly reduced but still very effective. On day 31 post first injection of the conjugates, the tumor volumes of the HM, HP4KM, and HP10KM groups with this dosage were 762, 556, and 454 mm^3^, respectively, and the corresponding tumor inhibition rates were 41.38%, 57.23%, and 65.07%, respectively.

In comparison, HP10KM had much higher in vivo antitumor activity than the other two conjugates. Compared with HM and HP4KM, HP10KM had the longest PEG chain modification and thus had the longest circulation half-life, which was 11.2 times and 4.5-fold longer than that of HM and HP4KM, respectively. However, it had reduced cytotoxicity at 22.5-fold and 3.5-fold lower than that of HM and HP4KM, respectively. The combined effect led to the relatively optimal in vivo tumor inhibition ability for HP10KM, which may indicate the importance of the circulation half-life of the conjugates for improving the tumor therapeutic ability of the conjugates, with better performance achieved even in the sacrifice of the cytotoxicity of the conjugates. Our results demonstrated that the introduction of a PEG chain was able to improve the therapeutic effect of ADC drugs based on miniaturized ligands; however, we need to adopt an appropriate length of the PEG chain for this modification and consider both the influence of the PEG chain on the half-life and the cytotoxic activity of the conjugates. This strategy may have good application potential in the development of antitumor drugs based on conjugates.

### 2.10. Liver Function and Immunohistochemical Analysis

The toxicity of these conjugates was assessed by liver function evaluation and immunohistochemical analysis of important tissues and organs of the mice that received treatment. For the liver function test, the serum levels of alanine aminotransferase (ALT) and aspartate aminotransferase (AST) in these mice were examined using kits. As shown in Figure 7E,F, all conjugates showed weak toxicity compared with the control group and the levels of ALT and AST were slightly increased in these groups. The AST level in the HP10KM group was significantly lower than that in the HM and HP4KM groups. Histological analysis of major organs, including the heart, liver, spleen, lung, kidney, and tumor tissues, showed that there was no obvious damage among the major organs after treatment with these conjugates (Figure 7G). These results indicate that these conjugates have good safety in treatment.

### 2.11. Off-Target Toxicity Analysis

The off-target toxicity of these conjugates was tested in BALB/c mice. The conjugates were i.v. injected into BALB/c mice at various dosages (2.0, 5.0, 10.0, 15.0, and 20.0 mg/kg), with free MMAE molecule as the control. As shown in Table 1, all mice died soon after injection of 5.0 mg/kg free MMAE; in the injection of 2.0 mg/kg free MMAE (not shown in Table 1), one mouse among the five died, and all other mice were blind and could not open their eyes. These data indicated that free MMAE molecules can cause serious side effects and even death in mice at dosages of 2.0 or 5.0 mg/kg. The conjugates had significantly increased tolerable dosages, and the mice obtained 100% survival rates at injection doses of 5.0 mg/kg (HM), 10.0 mg/kg (HP4KM), and 20.0 mg/kg (HP10KM). The maximum tolerable dosage (MTD) of HP10KM was at least 4-fold higher than that of free MMAE or HM. PEG modification, especially the 10 kDa modification, can significantly reduce the off-target toxicity of the conjugates in mice.

## 3. Materials and Methods

### 3.1. Reagents

MMAE and Gly3-VC-PAB-MMAE were obtained from Nanjing Levena Biopharma Co., Ltd. (Nanjing, China). Mal-PEG-NHS (4000 Da and 10,000 Da) was purchased from Beijing Jenkem Technology Co., Ltd. (Beijing, China). TCEP was obtained from TCI (Shanghai, China). The Live&Dead^TM^ Viability/Cytotoxicity Assay Kit was purchased from US Everbright^®^ Inc. (Suzhou, China). The APC Annexin V/PI Apoptosis Detection Kit was purchased from Biolegend (San Diego, CA, USA). All other materials were purchased from Tianjin Bestbay Biology Company (Tianjing, China).

### 3.2. Cell Lines and Animals

The human breast cancer cell lines BT-474 and MCF-7 were obtained from American Type Culture Collection (ATCC, USA). The human gastric cancer cell line NCI-N87 and prostatic carcinoma cell line PC-3 were purchased from the Chinese Academy of Sciences (Shanghai, China). BT-474 and NCI-N87 cells were cultured in RPMI 1640 medium, MCF-7 cells were cultured in DMEM, and PC-3 cells were cultured in F-12 culture medium. All media contained L-glutamine and were supplemented with 10% fetal bovine serum (FBS) and 1% penicillin-streptomycin.

BALB/c mice (female, 7 weeks old) and BALB/c nude mice (female, 6–8 weeks old) were purchased from Vital River Laboratory Animal Technology Co., Ltd. (Beijing, China). The studies were carried out in accordance with the guidelines of the Committee on Animals of Nankai University (Tianjin, China).

### 3.3. Cloning, Overexpression, and Purification of Affibody Z_HER2:2891_

The coding sequence for Z_HER2:2891_ was based on the amino acid sequence described by Feldwisch et al. [32]. A hexahistidyl (6 × His) tag was added at the N-terminus, and the sequence GGGGC containing a cysteine residue for drug conjugation was added at the C-terminus. The sequence was inserted into the pET28a vector between the NcoI and XhoI restriction sites. The obtained plasmid pET28a-Z_HER2:2891_ was transformed into BL21 (DE3) pLysS *Escherichia coli* cells and cultured in Luria–Bertani medium supplemented with 30 μg/mL kanamycin until the OD600 reached 0.6. Subsequently, the bacterial solution was induced by β-D-1-thiogalactopyranoside (IPTG) at a final concentration of 0.7 mM and incubated at 20 °C for 4–5 h with shaking before harvesting. The cell lysate was purified by a Ni-NTA agarose column (Roche, Indianapolis, IN, USA), and the eluent was dialyzed three times with reaction buffer (10 mM disodium hydrogen phosphate, 2.7 mM potassium chloride, 1.76 mM potassium dihydrogen phosphate, and 0.5 M NaCl, pH 7.4) to generate the affibody Z_HER2:2891_, which was verified by SDS-PAGE and mass spectrometry.

### 3.4. Preparation and Purification of the Conjugates

Before coupling, Z_HER2:2891_ was reduced with TCEP, the sulfhydryl group in the molecule is easily oxidized to form disulfide bonds in the process of purification and storage, which hinders the occurrence of coupling reactions. For reduction, Z_HER2:2891_ (0.5 mM in reaction buffer) was shaken with TCEP (1.1 equivalents with respect to Z_HER2:2891_) at room temperature for 1 h, and excess TCEP was removed by a PD-10 desalting column. The reduced Z_HER2:2891_ was used directly for the next coupling reaction.

For the preparation of HP4KM and HP10KM, bifunctional PEG4K or PEG10K was used. Here, 40 mM bifunctional PEG4K or PEG10K was first mixed with 80 mM Gly3-VC-PAB-MMAE (1.2 equivalents) and N,N-diisopropylethylamine (DIPEA, 2 equivalents) and shaken at room temperature for 2 h. Then, the mixture was reacted with the reduced Z_HER2:2891_ at an equivalent ratio of 1.1:1 (1.1 equivalents with respect to Z_HER2:2891_) and allowed to react at room temperature for 6 h. The products were purified through Ni-NTA purification resin and maintained in PBS buffer at −80 °C. For the conjugate HM, the bifunctional linker SMCC was used to replace the bifunctional PEG chains, and the coupling and purification strategies were the same as those of HP4KM and HP10KM.

### 3.5. MALDI-TOF-MS Analysis

A MALDI-TOF mass spectrometer (Bruker Daltonics) was used to analyze the mass of the conjugates and free affibody Z_HER2:2891_. For each sample, 1.5 μL protein solution was deposited on a well of a MALDI target plate. After evaporation, 1.5 μL HCCA matrix (4.0 mg/mL, dissolved in 50% CH_3_CN, 49.9% H_2_O, and 0.1% TFA) was added to the same well. Then, the dried matrix samples were analyzed with the mass spectrometer in the online positive mode with optimized instrument parameters.

### 3.6. HPLC Analysis

The identity of the conjugates was analyzed by HPLC using a C-4 column (250 × 4.6 mm) and a UV detector. The mobile phase was composed of A (0.14% TFA in H_2_O) and B (0.14% TFA in CH_3_CN). The column was initially held at A/B = 90/10 (*v*/*v*), and then, the concentration of B was constant at 10% in 5 min and 80% in 30 min. Subsequently, the concentration returned to 20% within another 10 min. Before each next injection, the column was allowed to equilibrate at A/B = 90/10 (*v*/*v*) for 30 min. Detection was followed at 220 nm.

### 3.7. Receptor Binding Analysis Based on ELISA

Human HER2 protein (ACRO Biosystems, Cat # HE2-H521y, Beijing, China) was plated in 96-well plates at 0.2 µg/well and incubated at 4 °C overnight. The plates were blocked with 3% bovine serum albumin (BSA) solution at room temperature for another 2 h and washed before adding gradient-diluted conjugates and incubating for another 2 h. The plates were then incubated with HRP bound mouse anti-His tag antibodies for 1 h at room temperature, washed and incubated with 100 μL/well of 3,3′,5,5′-tetramethylbenzidine (TMB) for 10 min before stopping the reaction with 2.0 N H_2_SO_4_ (100 µL/well), and the absorbance was determined at 450 nm. The data were analyzed by GraphPad Prism 6.0.

### 3.8. In Vivo Pharmacokinetics

ELISA was used to determine the half-life of the conjugates circulating in vivo. BALB/c mice (female, 7 weeks old) were injected with the conjugates at a dosage of 5.0 mg/kg per mouse via the tail vein. Blood samples were collected from the eye vein at different time points (3, 60, 120, 240, 360, 600, and 1440 min). The plasma was kept at room temperature for 30 min and then centrifuged at 4 °C to separate the serum. Human HER2 protein was coated on microtiter plates (5.0 mg/mL) and incubated at 4 °C for 18 h. The plates were washed with 0.1% PBS-T (PBS with 0.1% Tween-20) and blocked with 3% BSA solution for 1 h before adding serum samples diluted 1:400 with PBS buffer at 100 μL/well. After incubation for 1 h, the plates were washed with PBS-T again, incubated with mouse anti His-Tag antibody (1:2000 dilution, catalog No. 37-2900; Life Technologies, Camarillo, CA, USA) at room temperature for 1 h and with HRP-labeled goat anti-mouse IgG (1:2000 dilution, catalog No. CW0102; CWBio, Beijing, China) at room temperature for another 1 h. After washing with PBS-T, the plate was incubated with TMB for 15 min. The reaction was stopped with 100 μL 2.0 N sulfuric acid, and the absorbance was measured at 450 nm. The solution containing quantitative protein was used to draw the standard curve. GraphPad Prism 6.0 software was used to calculate the half-life of the conjugates.

### 3.9. Binding Selectivity Detected by Flow Cytometry

The binding affinity and selectivity of these conjugates with the HER2 receptor were analyzed with flow cytometry. NCI-N87 and BT474 cells with HER2 receptor positive expression and MCF-7 and PC-3 cells with HER2 receptor low expression were cultured in a 12-well plate at a cell density of 1.0 × 10^5^ cells/well at 37 °C for 24 h. Then, the cells were incubated with 50 nM conjugates or free Z_HER2:2891_ at 4 °C for 1 h, followed by incubation with FITC-labeled mouse anti-His IgG for another 1 h. After incubation and washing with PBS buffer, the cells were adjusted to a concentration of 1.0 × 10^6^ cells/mL and analyzed with a FACS Calibur instrument (BD Biosciences, San Jose, CA, USA), and FlowJo was used for data analysis.

### 3.10. In Vitro Cytotoxicity Assayed with MTT

Human gastric carcinoma NCI-N87 cells and human breast carcinoma BT-474 cells with HER2-high expression, human breast carcinoma MCF-7 cells with HER2-low expression and human prostatic carcinoma PC-3 cells with almost no HER2 expression were selected for this evaluation. The cancer cells were seeded in 96-well plates at a density of 5000 cells/well and cultured in a standard humidified cell incubator (37 °C, 5% CO_2_). After 24 h, various concentrations of the conjugates (2.56 pM to 1.0 µM) were added to the wells and cocultured at 37 °C for 72 h. Then, MTT solution (10 µL, 5.0 mg/mL, final concentration 0.5 mg/mL) was added and coincubated for another 4 h. After removing the supernatant, the methylenezinc crystal was dissolved in 100 μL dimethyl sulfoxide (DMSO), and the absorbance was measured at 490 nm. GraphPad Prism software (GraphPad Software Inc., San Diego, CA, USA) was used for the data analysis.

To further verify the role of the HER2 receptor in the cytotoxicity of these conjugates, a competitive inhibition assay was used. Here, HER2-high-expressing NCI-N87 and BT-474 cells (5000 cells/well) inoculated in 96-well plates were first preincubated with 50 μM free Z_HER2:2891_ protein for 2 h to block the HER2 receptor and then incubated with various concentrations of the conjugates for 72 h. Cell viability was measured with MTT reagents in a similar way.

### 3.11. Visual Observation of In Vitro Cytotoxicity through Live/Dead Staining

HER2-positive NCI-N87 cells and HER2-negative PC-3 cells were plated in 96-well plates at a cell density of 5000 cells/well and cultured at 37 °C for 24 h. Then, 200 nM HM, HP4KM, HP10KM and MMAE were added to the wells and coincubated at 37 °C for another 72 h. After washing with PBS buffer, the cells were stained with calcein-AM, ethidium homodimer-1 (EthD-1), and PBS at a ratio of 1:4:1000 for 15 min and then imaged under an inverted microscope (DMI 4000B, Leica, USA).

### 3.12. Cell Apoptosis Assay with the APC Annexin V/PI Apoptosis Detection Kit

HER2-positive NCI-N87 cells and HER2-negative PC-3 cells plated in 12-well plates at a density of 1.0 × 10^5^ cells/well were cultured at 37 °C for 24 h and then coincubated with 200 nM HM, HP4KM, HP10KM, and MMAE for another 24, 48, and 72 h. The cells were digested and washed with AV binding buffer, and then the cell concentration was adjusted to 1.0 × 10^6^ cells/mL. APC annexin V and PI were added and stained at room temperature for 20 min, and the stained cells were analyzed by flow cytometry.

### 3.13. Selective Induction of G2 Growth Arrest Analyzed via Flow Cytometry

NCI-N87 cells were plated at a density of 2.0 × 10^5^ cells per well in a 12-well plate. After culture for 24 h, the cells were incubated with 1.0 µM of the conjugates or free MMAE for 16 h at 37 °C. PBS was added as the control group. Then, the cells were collected, fixed with ice-cold ethanol (75% *v*/*v*) for 16 h, washed with PBS twice and stained with 50 μg/mL propidium iodide (PI) solution in PBS buffer containing 0.1% Triton X-100 along with DNase-free RNase A at 37 °C for 30 min in the dark. Then, the cells were collected and analyzed via flow cytometry (BD FACS Calibur), and ModFit software was used for the data analysis.

### 3.14. In Vivo Antitumor Activity

A HER2-positive NCI-N87 xenograft mouse model was adopted to evaluate the in vivo antitumor activity of these conjugates. The model was established by subcutaneous inoculation of 5.0 × 10^6^ NCI-N87 cells per mouse in the right flanks of female BALB/c nude mice. When the tumor volume reached approximately 210 mm^3^, the mice were randomized into four groups (*n* = 5) and injected with various dosages of the conjugates once every three days for a total of four times, and PBS injection was used as the control. The tumor size and weight were monitored every three days until day 31. The tumor dimensions were measured with a digital caliper, and their volumes were calculated using the formula length × (width)^2^/2.

On day 31 post first injection of the conjugates, blood samples (200 µL) were collected from the mice receiving treatment with a 1.5 mg/kg dose injection and the serum was separated by centrifugation at 3000 rpm for 10 min. The levels of ALT and AST in serum were quantified by ALT and AST activity assay kits (Nanjing Jiancheng Bioengineering Institute), respectively. The mice were then euthanized, and their tissues and organs were collected and fixed with 4% paraformaldehyde. The samples were embedded in paraffin, stained with hematoxylin and eosin (H&E), and visualized by a Nikon eclipse E100 microscope for histopathological analysis.

### 3.15. Off-Target Toxicity Analysis

BALB/c mice (*n* = 5) were i.v. injected with the indicated amount of the conjugates or free MAEE (2.0, 5.0, 10.0, 15.0, and 20.0 mg/kg). The moribund conditions and death events of the mice were detected and reported within two weeks post injection.

### 3.16. Statistical Analysis

All data are presented as the mean ± SEM (standard error of the mean). Differences between the values of two groups were evaluated by two-tailed Student’s *t*-test in GraphPad Prism 6.0 software. A *p*-value < 0.05 was considered significant.

## 4. Conclusions

In this study, we proposed a new method for designing and synthesizing miniaturized ADC molecules. The drug molecule and affibody protein were linked through a bifunctional PEG chain, and site-specific coupling was achieved by introduction of an extra cysteine residue into the protein. The inserted PEG chains significantly prolonged the circulation half-life but also obviously reduced the cytotoxicity of the conjugates, with 2.5- and 11.2-fold half-life extensions and 4.5- and 22-fold in vitro cytotoxicity reductions by 4 and 10 kDa PEG modification, respectively. The combined effect led to HP10KM having the best tumor therapeutic ability in the NCI-N87 tumor model, and its off-target toxicity was 4 times lower than that of HM. These results demonstrate that PEG chain modification and circulation half-life extension can significantly improve the tumor therapeutic ability of the conjugates. However, they may also indicate that it is important to use PEG chains of appropriate length for this modification. Moreover, the effects of PEG chains on the half-life and cytotoxicity of the conjugates need to be considered in combination. These results may provide guidance for the development of ADC drugs based on miniaturized ligands.

## Figures and Tables

**Table 1:** In vivo off-target toxicity of the conjugates and free monomethyl auristatin E (MMAE).

Dead/Total Mice				
Inject Dose	5.0 mg/kg	10.0 mg/kg	15.0 mg/kg	20.0 mg/kg
HM	0/5	3/5	-	-
HP4KM	-	0/5	1/5	4/5
HP10KM	-	-	0/5	0/5
MMAE	5/5	-	-	-

## Data Availability

Not applicable.

## Supplementary Materials

The following are available online at https://www.mdpi.com/1422-0067/22/4/1540/s1.
