# Supplementary material for: PEG Linker Improves Antitumor Efficacy and Safety of Affibody-Based Drug Conjugates
Source: Int J Mol Sci. 2021 Feb 3;22(4):1540. doi: 10.3390/ijms22041540 (PMC7913616; doi:10.3390/ijms22041540)
Supplement: Supplementary file 1 [file ijms-22-01540-s001.pdf]

Supporting Information

PEG Linker Improves Antitumor Efficacy and Safety of Affibody-based Drug Conjugates

Qiyu Li [1,†], Wenjing Li [1,†], Keyuan Xu [1], Yutong Xing [1], Haobo Shi [1], Zhe Jing [1], Shuang Li [1,*] and Zhangyong Hong [1,*]

[1] State Key Laboratory of Medicinal Chemical Biology, College of Life Sciences, Nankai University,Tianjin 300071, China; 1047244563@qq.com (Q.L.); L800450@163.com (W.L.); 2574070637@qq.com (K.X.); 942220098@qq.com (Y.X.); bohaoshi@qq.com (H.S.); 274298803@qq.com (Z.J.);

* Correspondence: lishuang5258@163.com (S.L.); Hongzy@nankai.edu.cn (Z.H.);

* Tel.: +86-022-23498707 (S.L.); +86-022-23498707 (Z.H.)

† These authors contributed equally to the project.

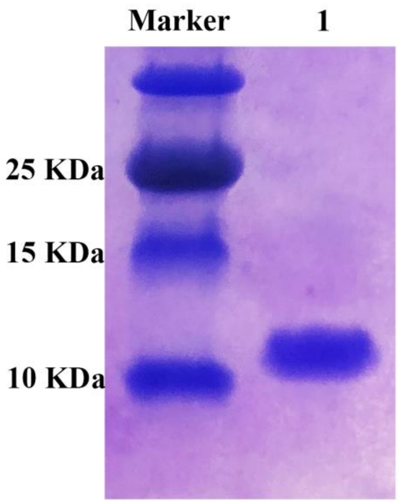

Figure S1. SDS-PAGE analysis of affibody $Z_{HER2:2891}$. The purified affibody $Z_{HER2:2891}$ was loaded onto a 16% polyacrylamide gel with protein weight standards and stained with Coomassie brilliant blue.

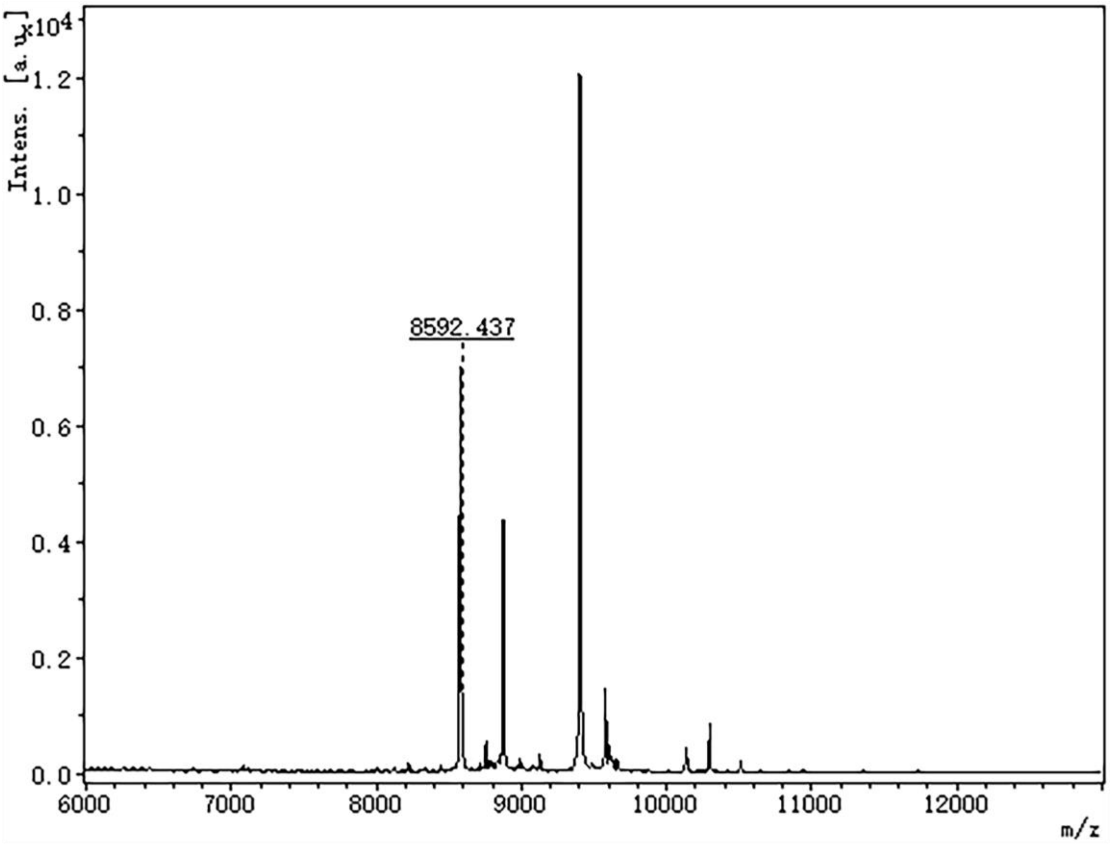

Figure S2. MALDI-TOF-MS analysis of $Z_{HER2:2891}$. Theoretical molecular mass is 8588.23 Da, and detected molecular mass is 8592.44 Da.

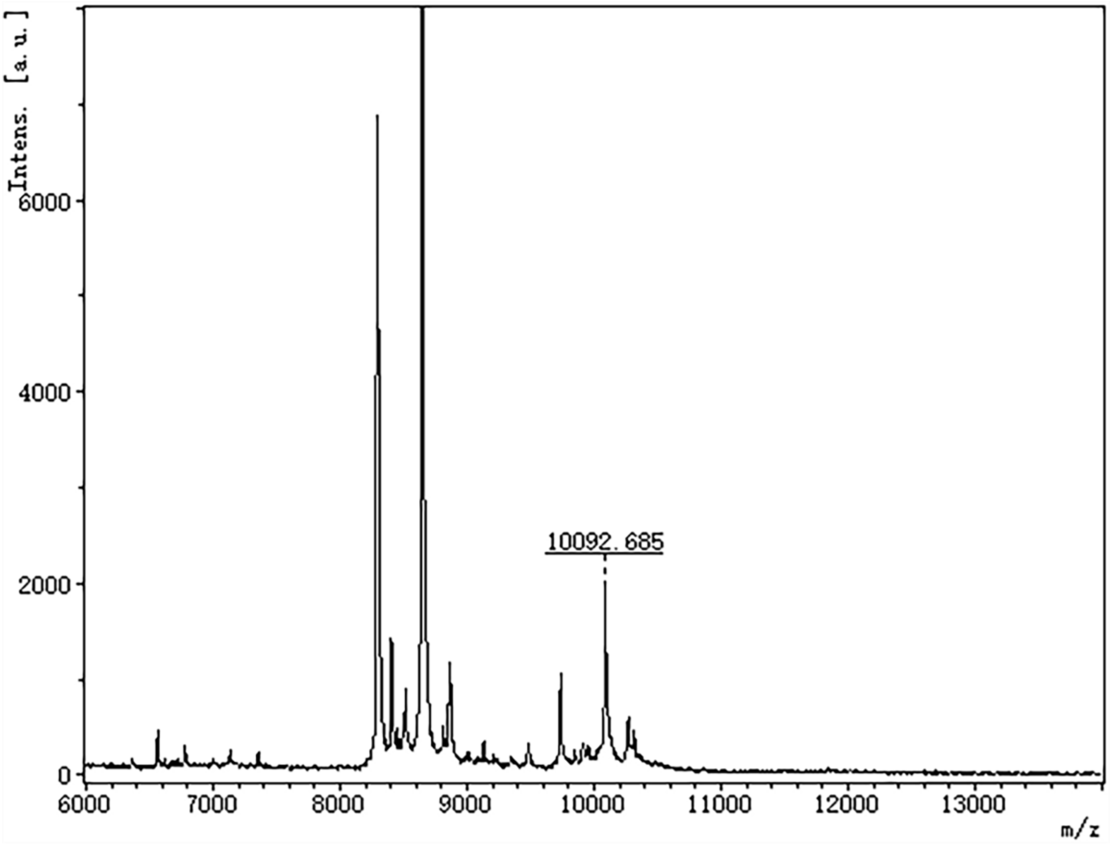

Figure S3. MALDI-TOF-MS analysis of purified HM. Theoretical molecular mass is 10101.1 Da, and detected molecular mass is 10092.69 Da.

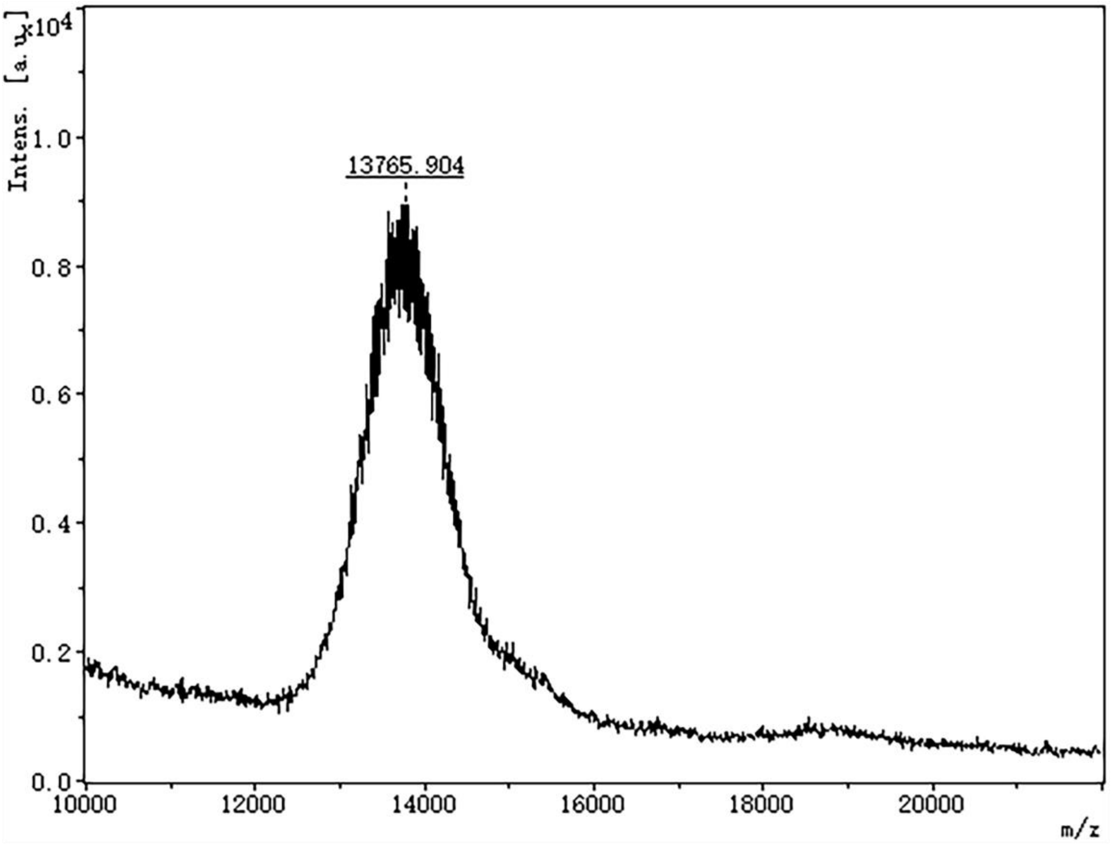

Figure S4. MALDI-TOF-MS analysis of purified HP4KM. Theoretical molecular mass is 13782.97 Da, and detected molecular mass is 13765.90 Da.

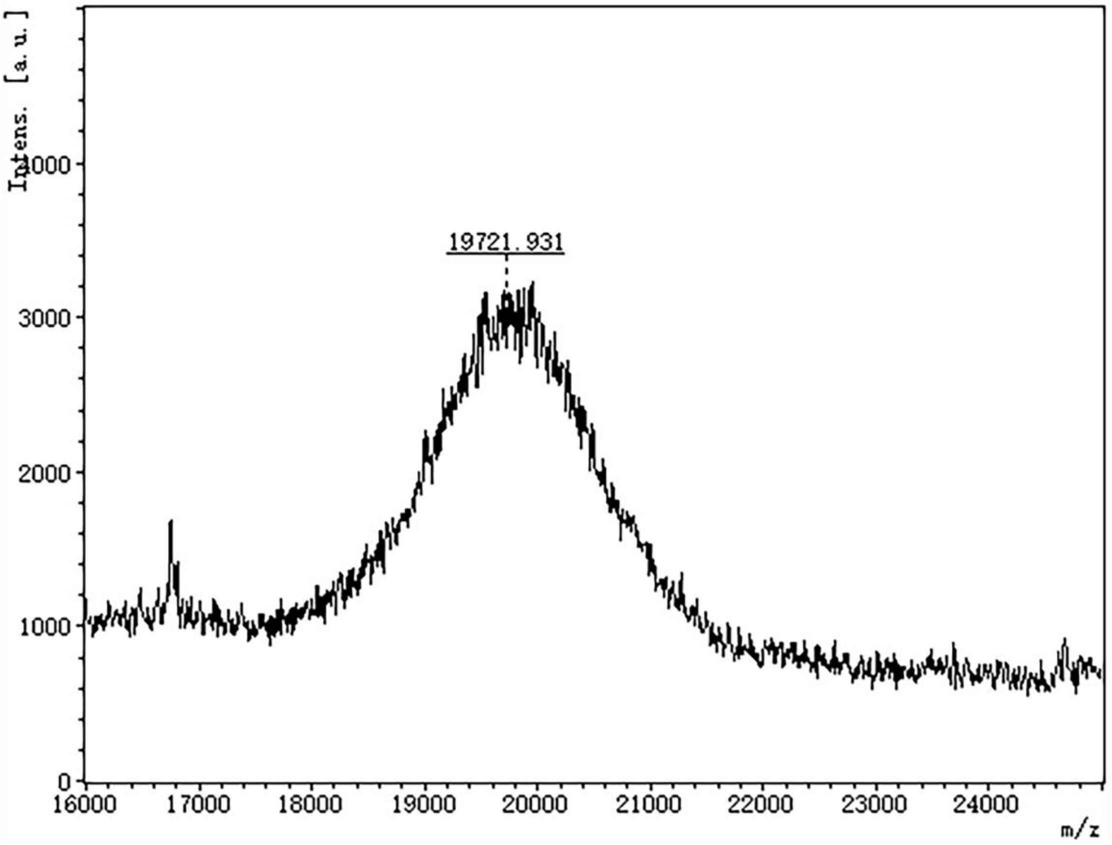

Figure S5. MALDI-TOF-MS analysis of purified HP10KM. Theoretical molecular mass is 19782.97 Da, and detected molecular mass is 19721.93 Da.

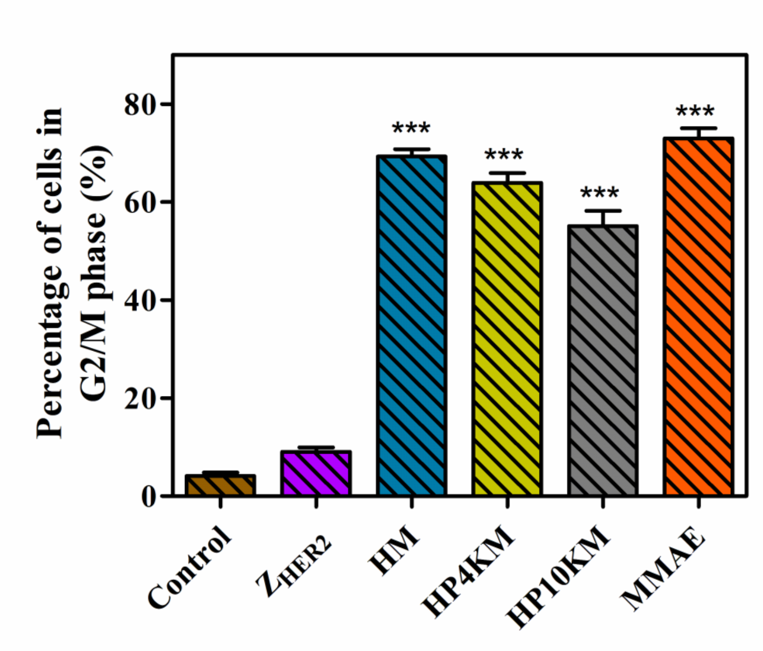

Figure S6. Percentage of NCI-N87 cells in G2/M phase after treatment with the conjugates at a dose of 1.0 μM for 16 h (n = 3). *p < 0.05, **p < 0.01, ***p < 0.001.
